# Supplementary material for: Phosphorylation disrupts long-distance electron transport in cytochrome c
Source: Nat Commun. 2022 Nov 19;13:7100. doi: 10.1038/s41467-022-34809-1 (PMC9675734; doi:10.1038/s41467-022-34809-1)
Supplement: Supplementary file 1 — Supplementary Information [file 41467_2022_34809_MOESM1_ESM.pdf]

Supplementary Information

Phosphorylation disrupts long-distance electron transport in cytochrome *c*

Alexandre M. J. Gomila[1,2,‡], Gonzalo Pérez-Mejías[3,‡], Alba Nin-Hill[4,‡], Alejandra Guerra-Castellano[3], Laura Casas-Ferrer[1,8], Sthefany Ortiz-Tescari[1], Antonio Díaz-Quintana[3], Josep Samitier[1,2,5], Carme Rovira[4,6,*], Miguel A. De la Rosa[3], Irene Díaz-Moreno[3,*], Pau Gorostiza[1,2,6,*], Marina I. Giannotti[1,2,7,*], Anna Lagunas[1,2,*].

[1]Institute for Bioengineering of Catalonia (IBEC), The Barcelona Institute for Science and Technology (BIST), Barcelona, Spain.

[2]CIBER-BBN, ISCIII, Barcelona, Spain.

[3]Institute for Chemical Research–cicCartuja, Universidad de Sevilla, Consejo Superior de Investigaciones Científicas (CSIC), Sevilla, Spain.

[4]University of Barcelona, Department of Inorganic and Organic Chemistry, Institute of Theoretical Chemistry (IQTCUB), Barcelona, Spain.

[5]Department of Electronics and Biomedical Engineering, University of Barcelona (UB), Faculty of Physics, Barcelona, Spain.

[6]Catalan Institution for Research and Advanced Studies (ICREA), Barcelona, Spain.

[7]Department of Materials Science and Physical Chemistry, University of Barcelona (UB), Faculty of Chemistry, Barcelona, Spain.

[8]Present address: Laboratoire Charles Coulomb (L2C), UMR 5221 CNRS-Université de Montpellier, France.

[‡]These authors contributed equally.

[*]corresponding authors: c.rovira@ub.edu, idiazmoreno@us.es, pau@icrea.cat, migiannotti@ibecbarcelona.eu, alagunas@ibecbarcelona.eu

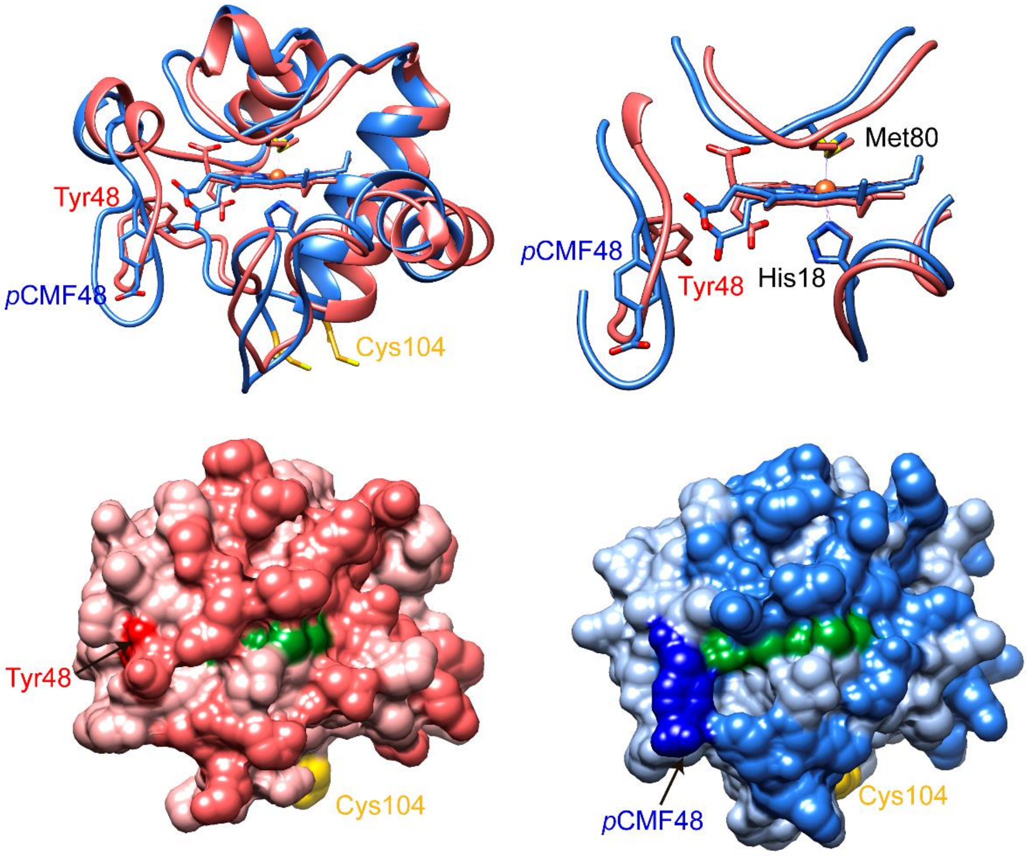

Supplementary Figure 1. Effect of tyrosine phosphorylation at position 48 on the structure of human cytochrome *c*. (**Upper**) *Left:* Superimposition of ribbon representations for wild-type C*c* (red, PDB: 2N9I[1]) and its Y48*p*CMF variant (blue, PDB: 2N3Y[2]) in the reduced state. Location of Cys104—used for immobilization on Au(111) surface—is marked in yellow. *Right*: detailed view of the heme group, iron axial ligands (Met80 and His18) and Tyr48/*p*CMF48 residues, highlighting how phosphorylation induces drastic structural changes at the heme environment and tyrosine-harboring loop. (**Lower**) Surface representation of wild-type C*c* (left, red) and its Y48*p*CMF variant (right, blue), with color ranging from lighter to darker for every residue depending on the intensity of their interaction with C$c_{1,}$ as determined by NMR spectroscopy.[2] The conformational adjustment induced by tyrosine phosphorylation enhances the heme group (green) solvent accessibility. The pictures were created with the UCFS Chimera 1.15rc software.[3]

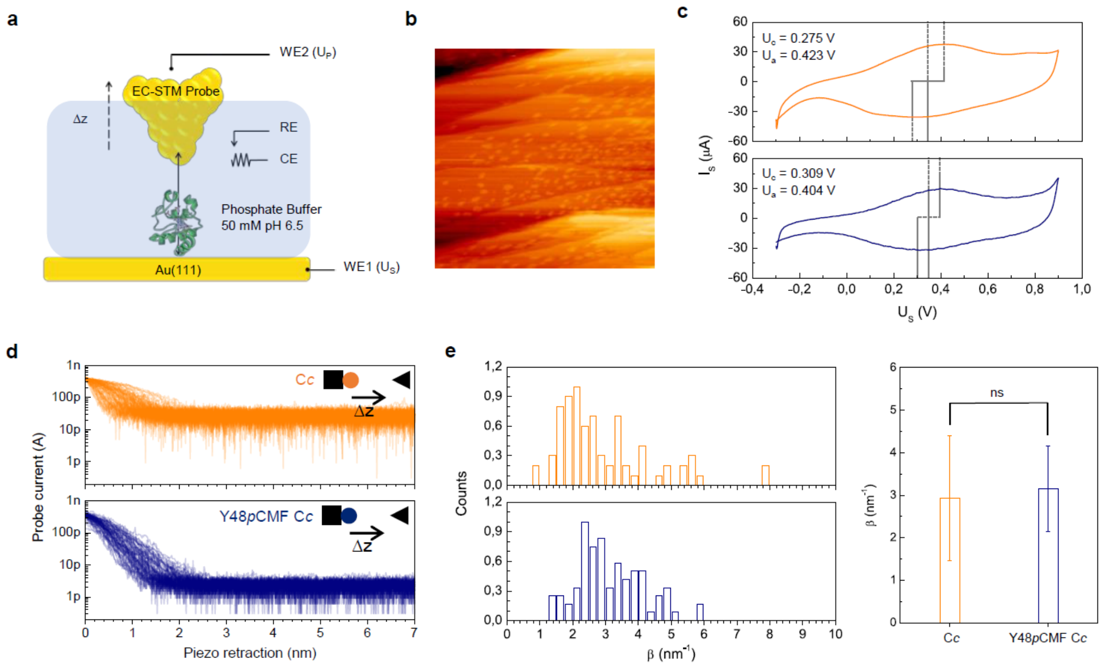

Supplementary Figure 2. a, Schematics of the experimental set-up for current-distance (*I*-*z*) spectroscopy studies of cytochrome *c* (C*c*) under bipotentiostatic control in near to physiological environment in EC-STM. Abbreviations: WE, working electrode; RE, reference electrode; CE, counter electrode. **b,** EC-STM image of 400x400 nm of C*c* immobilized on Au(111) electrode through the C-terminal mutation E104C. Current set point = 0.3 nA. **c,** Cyclic voltammetry of Au(111) electrodes modified with C*c* (orange) and Y48*p*CMF C*c* (navy). The corresponding reduction (U_c) an oxidation (U_a) peaks for the Fe^{3+}/Fe^{2+} couple are indicated. **d,** Ensemble of semi-logarithmic current-distance (*I*-*z*) curves obtained during probe retraction (15 nm from the set point at 12 nm/s) for C*c* (orange) and Y48*p*CMF C*c* (navy). Current set point = 0.4 nA. **e,** Histograms of distance decay factors (β) quantified from individual curves in **d**, and the corresponding plot of the averaged β values from gaussian fit (mean ± s.d.) showing no statistically significant differences (two sample t-test, t Statistic = 1.111, degrees of freedom = 130.0, $P < 0.05$). $\beta = 3 \pm 1$ nm^{-1} for both C*c* (n = 75 *I-z* curves from 2 independent experiments) and Y48*p*CMF C*c* (n = 82 *I-z* curves from 2 independent experiments). All the experiments were performed in 50 mM phosphate buffer, pH 6.5. Us = -200 mV and at constant bias of 800 mV. Source data are provided as a Source Data file.

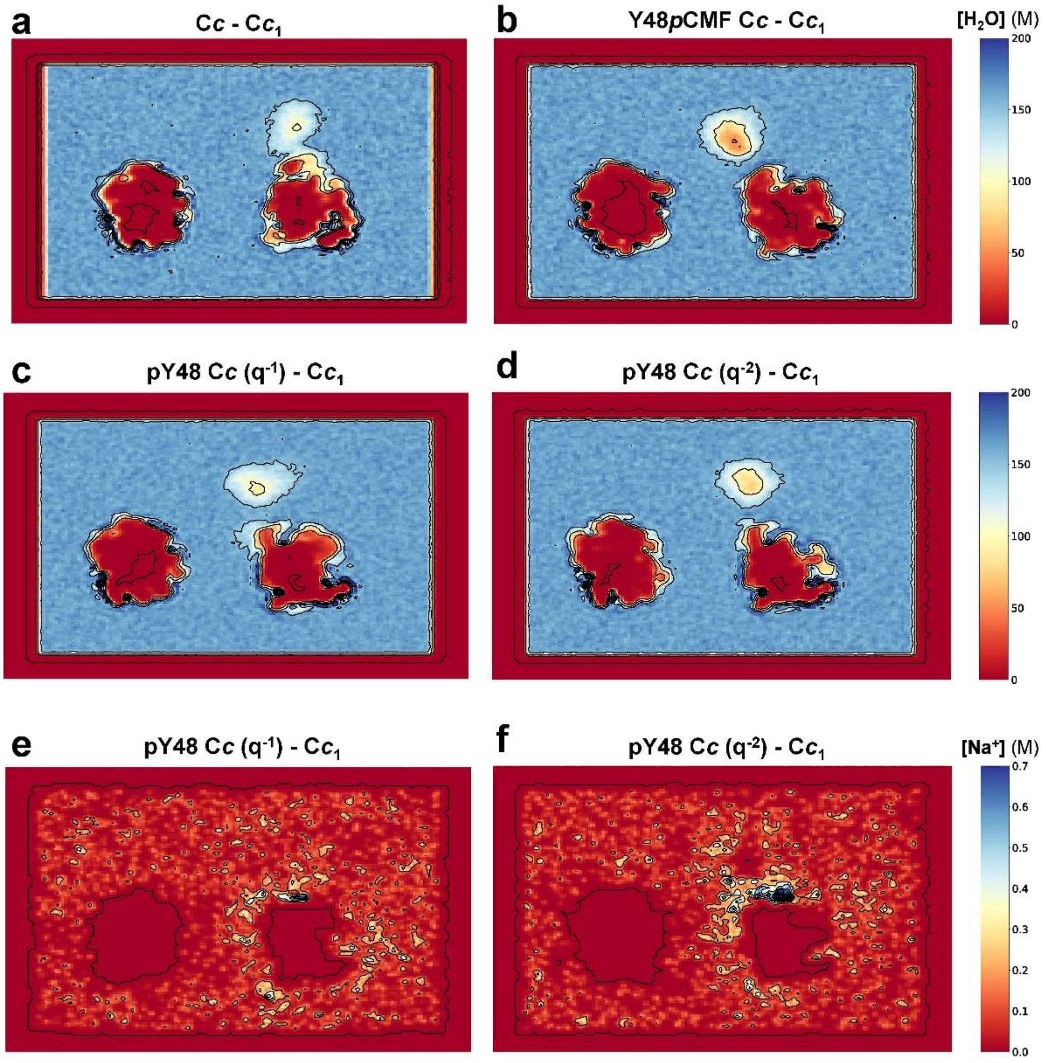

Supplementary Figure 3. MD simulations side view of the average water concentration (M) map at the X-axe plane dissecting both Cc_1-C*c* redox active sites facing each other, set 3.2 nm apart taking the most external hydrogen atom from the -CH_3 distal pairs from each heme group, that is 4.2 nm taking the Fe-to-Fe distance, in the aqueous medium, at the same plane as Figure 4 of the main text. **(a)** Cc_1-C*c*, **(b)** Cc_1-Y48*p*CMF C*c in silico*, **(c)**, Cc_1-pY48 C*c* (O-phospho-L-tyrosine) with charge –1, **(d)** Cc_1-pY48 C*c* (O-phospho-L-tyrosine) with charge –2. Each contour line displays an increase of 50 units in concentration. No major differences in water concentration are observed and only a mild higher mobility of the phosphorylated C*c* is perceived. Y48*p*CMF C*c* displays a more accessible surface for water molecules due to a more flexible structure. **(e, f)** Side view of the averaged sodium concentration (M) map at the X-axe plane dissecting both Cc_1 – C*c* redox-active sites (pY48 C*c* q^{-1} **(e)** and pY48 C*c* q^{-2} **(f)**, respectively) facing each other as described. Each contour line displays an increase of 0.15 M. Source data are provided as a Source Data file.

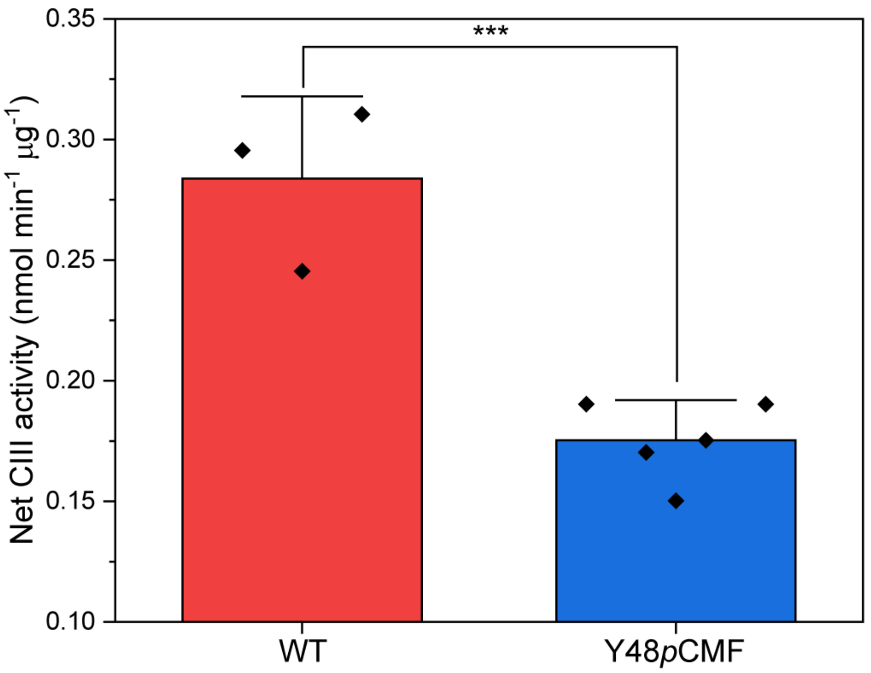

Supplementary Figure 4. Complex III activity with WT or Y48*p*CMF C*c* as the electron acceptor. Activities were measured by monitoring the absorption changes at 550 nm and reported as mean ± s.d. (n=3 and 5 independent experiments for WT and Y48*p*CMF, respectively), showing significant differences (two sample t-test were used; t Statistic=6.22, degrees of freedom=6.00, ***P=4.00x10^{-4}). Source data are provided as a Source Data file.

Supplementary References

1. Imai, M. *et al.* Investigation of the redox-dependent modulation of structure and dynamics in human cytochrome c. *Biochem. Biophys. Res. Commun.* **469**: 978–984 (2016).

2. Moreno-Beltrán, B. *et al.* Structural basis of mitochondrial dysfunction in response to cytochrome c phosphorylation at tyrosine 48. *Proc. Natl. Acad. Sci.* **114**: E3041-E3050 (2017).

3. Pettersen, E. F. *et al.* UCSF Chimera—A visualization system for exploratory research and analysis. *J. Comput. Chem.* **25**: 1605–1612 (2004).
